# *N*,4-Dimethyl-*N*-(4-nitro­benz­yl)benzene­sulfonamide

**DOI:** 10.1107/S1600536808000330

**Published:** 2008-02-15

**Authors:** Hong-Yuan Zhu, Zhou Wu, Shende Jiang

**Affiliations:** a School of Pharmaceutical Science and Technology, Tianjin University, Tianjin 300072, People’s Republic of China

## Abstract

In the title compound, C_15_H_16_N_2_O_4_S, there is a dihedral angle of 63.30 (8)° between the nitro­benzyl and benzene rings, which are separated by a sulfonamide unit The crystal packing is stabilized by a C—H⋯O inter­action.

## Related literature

For the use of aromatic nitro and amine compounds as precursors in dye synthesis, see: Lauwiner *et al.* (1998 ▶); Yang *et al.* (2004 ▶). For the preparation of the title compound, see: Andersen *et al.* (1988 ▶). For bond-length data, see: Allen *et al.* (1987 ▶).
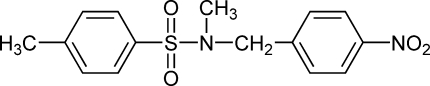


## Experimental

### 

#### Crystal data

C_15_H_16_N_2_O_4_S
*M* *_r_* = 320.36
Monoclinic, 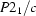

*a* = 9.5694 (19) Å
*b* = 6.1335 (12) Å
*c* = 26.126 (5) Å
β = 100.03 (3)°
*V* = 1510.0 (5) Å^3^
*Z* = 4
Mo *K*α radiation
μ = 0.23 mm^−1^
*T* = 113 (2) K
0.20 × 0.18 × 0.10 mm

#### Data collection

Rigaku Saturn diffractometer
Absorption correction: multi-scan (*CrystalClear*; Rigaku, 2003 ▶) *T*_min_ = 0.955, *T*_max_ = 0.977
8935 measured reflections
2661 independent reflections
2188 reflections with *I* > 2σ(*I*)
*R*_int_ = 0.057

#### Refinement

*R*[*F* ^2^ > 2σ(*F* ^2^)] = 0.057
*wR*(*F* ^2^) = 0.123
*S* = 1.10
2661 reflections
201 parameters
H-atom parameters constrained
Δρ_max_ = 0.36 e Å^−3^
Δρ_min_ = −0.45 e Å^−3^

### 

Data collection: *CrystalClear* (Rigaku, 2003 ▶); cell refinement: *CrystalClear*; data reduction: *CrystalClear*; program(s) used to solve structure: *SHELXS97* (Sheldrick, 2008 ▶); program(s) used to refine structure: *SHELXL97* (Sheldrick, 2008 ▶); molecular graphics: *SHELXTL* (Bruker, 1997 ▶); software used to prepare material for publication: *SHELXTL*.

## Supplementary Material

Crystal structure: contains datablocks global, I. DOI: 10.1107/S1600536808000330/sj2454sup1.cif

Structure factors: contains datablocks I. DOI: 10.1107/S1600536808000330/sj2454Isup2.hkl

Additional supplementary materials:  crystallographic information; 3D view; checkCIF report

## Figures and Tables

**Table 1:** Hydrogen-bond geometry (Å, °)

*D*—H⋯*A*	*D*—H	H⋯*A*	*D*⋯*A*	*D*—H⋯*A*
C9—H9*A*⋯O1^i^	0.99	2.32	3.287 (4)	166

Symmetry code: (i) 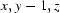.

**Fig. 1.**
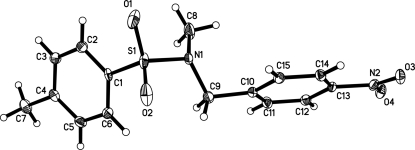
The structure of (I) with displacement ellipsoids drawn at the 30% probability level and H atoms shown as small spheres of arbitrary radii.

**Fig. 2.**
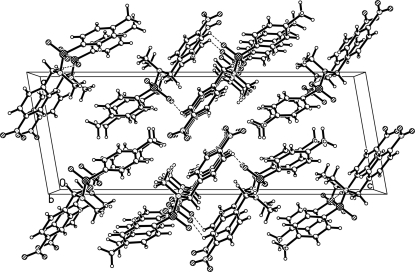
The crystal packing of (I) with hydrogen bonds drawn as dashed lines.

## supplementary crystallographic information

### Comment

Aromatic amines are widely employed for organic synthesis, especially as dye intermediates. One method for preparing aromatic amines is reduction of the corresponding aromatic nitro compound (Lauwiner *et al.*, 1998). In our recent work, the title compound (I), Fig. 1, was reduced to the corresponding aromatic amine for potential use as an intermediate in the synthesis of Acid Blue 264 dye according to Yang *et al.* (2004).

In (I), all bonds lengths and angles are normal (Allen *et al.*, 1987). The dihedral angle between the two aryl rings is 63.30 (8) Å. The distances of S1—C1 and S1—N1 are 1.762 (3) and 1.637 (2) Å respectively. The neighboring molecules are linked together by weak C—H···O hydrogen bonds, Table 1.

### Experimental

The title compound (I) was synthesized according to the procedure of Andersen *et al.* (1988). Colorless single crystals (m.p. 403–404 K) were obtained by slow evaporation of a solution in absolute alcohol.

### Refinement

C-bound H atoms were positioned geometrically and refined in the riding-model approximation, with d(C—H) = 0.93 Å, *U*_iso_=1.2*U*_eq_ (C) for aromatic, 0.96 Å, *U*_iso_ = 1.5*U*_eq_ (C) for CH_3_ atoms and 0.97 Å, *U*_iso_ = 1.2*U*_eq_ (C) for CH_2_ atoms.

### Crystal data

**Table:**

C_15_H_16_N_2_O_4_S	*F*_000_ = 672
*M**_r_* = 320.36	*D*_x_ = 1.409 Mg m^−^^3^
Monoclinic, *P*2_1_/*c*	Melting point = 403–404 K
Hall symbol: -P 2ybc	Mo *K*α radiation λ = 0.71073 Å
*a* = 9.5694 (19) Å	Cell parameters from 3609 reflections
*b* = 6.1335 (12) Å	θ = 2.2–27.9º
*c* = 26.126 (5) Å	µ = 0.23 mm^−^^1^
β = 100.03 (3)º	*T* = 113 (2) K
*V* = 1510.0 (5) Å^3^	Block, colorless
*Z* = 4	0.20 × 0.18 × 0.10 mm

### Data collection

**Table:**

Rigaku Saturn diffractometer	2661 independent reflections
Radiation source: fine-focus sealed tube	2188 reflections with *I* > 2σ(*I*)
Monochromator: graphite	*R*_int_ = 0.057
Detector resolution: 7.31 pixels mm^-1^	θ_max_ = 25.0º
*T* = 113(2) K	θ_min_ = 2.2º
ω scans	*h* = −11→11
Absorption correction: multi-scan(CrystalClear; Rigaku, 2003)	*k* = −6→7
*T*_min_ = 0.955, *T*_max_ = 0.977	*l* = −31→26
8935 measured reflections	

### Refinement

**Table:**

Refinement on *F*^2^	Secondary atom site location: difference Fourier map
Least-squares matrix: full	Hydrogen site location: inferred from neighbouring sites
*R*[*F*^2^ > 2σ(*F*^2^)] = 0.057	H-atom parameters constrained
*wR*(*F*^2^) = 0.123	*w* = 1/[σ^2^(*F*_o_^2^) + (0.0454*P*)^2^ + 1.1085*P*] where *P* = (*F*_o_^2^ + 2*F*_c_^2^)/3
*S* = 1.10	(Δ/σ)_max_ = 0.001
2661 reflections	Δρ_max_ = 0.36 e Å^−^^3^
201 parameters	Δρ_min_ = −0.45 e Å^−^^3^
Primary atom site location: structure-invariant direct methods	Extinction correction: none

### Special details

**Table:**

Geometry. All e.s.d.'s (except the e.s.d. in the dihedral angle between two l.s. planes) are estimated using the full covariance matrix. The cell e.s.d.'s are taken into account individually in the estimation of e.s.d.'s in distances, angles and torsion angles; correlations between e.s.d.'s in cell parameters are only used when they are defined by crystal symmetry. An approximate (isotropic) treatment of cell e.s.d.'s is used for estimating e.s.d.'s involving l.s. planes.
Refinement. Refinement of *F*^2^ against ALL reflections. The weighted *R*-factor *wR* and goodness of fit *S* are based on *F*^2^, conventional *R*-factors *R* are based on *F*, with *F* set to zero for negative *F*^2^. The threshold expression of *F*^2^ > σ(*F*^2^) is used only for calculating *R*-factors(gt) *etc*. and is not relevant to the choice of reflections for refinement. *R*-factors based on *F*^2^ are statistically about twice as large as those based on *F*, and *R*- factors based on ALL data will be even larger.

### Fractional atomic coordinates and isotropic or equivalent isotropic displacement parameters (Å^2^)

**Table:**

	*x*	*y*	*z*	*U*_iso_*/*U*_eq_	
S1	0.12405 (8)	0.46188 (11)	0.13266 (3)	0.0293 (2)	
O1	0.0775 (3)	0.6688 (3)	0.14746 (9)	0.0531 (7)	
O2	0.2167 (2)	0.4497 (4)	0.09498 (8)	0.0445 (6)	
O3	−0.5173 (2)	−0.0352 (3)	−0.10469 (8)	0.0334 (5)	
O4	−0.5383 (2)	−0.3199 (3)	−0.05751 (8)	0.0329 (5)	
N1	−0.0187 (2)	0.3232 (3)	0.10887 (9)	0.0221 (5)	
N2	−0.4801 (2)	−0.1489 (4)	−0.06589 (9)	0.0259 (5)	
C1	0.2079 (3)	0.3328 (4)	0.19015 (11)	0.0210 (6)	
C2	0.2046 (3)	0.4289 (5)	0.23762 (12)	0.0316 (7)	
H2	0.1530	0.5601	0.2395	0.038*	
C3	0.2765 (3)	0.3336 (5)	0.28224 (12)	0.0311 (7)	
H3	0.2738	0.4004	0.3149	0.037*	
C4	0.3529 (3)	0.1419 (4)	0.28053 (11)	0.0251 (6)	
C5	0.3516 (3)	0.0458 (4)	0.23231 (12)	0.0285 (7)	
H5	0.4007	−0.0878	0.2304	0.034*	
C6	0.2809 (3)	0.1393 (4)	0.18703 (11)	0.0264 (6)	
H6	0.2823	0.0723	0.1543	0.032*	
C7	0.4382 (3)	0.0457 (5)	0.32894 (12)	0.0364 (8)	
H7A	0.5367	0.0959	0.3327	0.055*	
H7B	0.4356	−0.1137	0.3265	0.055*	
H7C	0.3981	0.0922	0.3592	0.055*	
C8	−0.1208 (3)	0.2999 (7)	0.14427 (14)	0.0496 (10)	
H8A	−0.2139	0.2607	0.1244	0.074*	
H8B	−0.1282	0.4381	0.1624	0.074*	
H8C	−0.0886	0.1852	0.1697	0.074*	
C9	0.0035 (3)	0.1211 (4)	0.08077 (12)	0.0282 (7)	
H9A	0.0301	0.0020	0.1062	0.034*	
H9B	0.0831	0.1428	0.0616	0.034*	
C10	−0.1269 (3)	0.0558 (4)	0.04315 (11)	0.0222 (6)	
C11	−0.1885 (3)	−0.1471 (4)	0.04786 (11)	0.0259 (6)	
H11	−0.1499	−0.2403	0.0758	0.031*	
C12	−0.3050 (3)	−0.2153 (4)	0.01256 (11)	0.0235 (6)	
H12	−0.3473	−0.3534	0.0159	0.028*	
C13	−0.3577 (3)	−0.0763 (4)	−0.02765 (11)	0.0215 (6)	
C14	−0.2999 (3)	0.1272 (4)	−0.03363 (11)	0.0231 (6)	
H14	−0.3386	0.2195	−0.0617	0.028*	
C15	−0.1842 (3)	0.1921 (4)	0.00249 (11)	0.0223 (6)	
H15	−0.1435	0.3317	−0.0006	0.027*	

### Atomic displacement parameters (Å^2^)

**Table:**

	*U*^11^	*U*^22^	*U*^33^	*U*^12^	*U*^13^	*U*^23^
S1	0.0358 (4)	0.0223 (4)	0.0238 (4)	−0.0080 (3)	−0.0117 (3)	0.0051 (3)
O1	0.0816 (18)	0.0182 (11)	0.0437 (16)	0.0046 (10)	−0.0330 (13)	−0.0008 (9)
O2	0.0393 (12)	0.0675 (16)	0.0242 (13)	−0.0274 (11)	−0.0015 (10)	0.0159 (11)
O3	0.0316 (11)	0.0426 (12)	0.0224 (12)	−0.0005 (9)	−0.0054 (9)	0.0013 (9)
O4	0.0290 (11)	0.0425 (12)	0.0269 (12)	−0.0163 (9)	0.0041 (9)	−0.0049 (9)
N1	0.0191 (11)	0.0236 (12)	0.0210 (14)	−0.0015 (9)	−0.0036 (10)	−0.0023 (9)
N2	0.0232 (12)	0.0347 (14)	0.0199 (14)	−0.0022 (10)	0.0039 (10)	−0.0082 (11)
C1	0.0172 (13)	0.0232 (14)	0.0192 (15)	−0.0034 (10)	−0.0060 (11)	0.0046 (11)
C2	0.0344 (16)	0.0269 (15)	0.0291 (18)	0.0094 (12)	−0.0067 (14)	−0.0033 (12)
C3	0.0319 (16)	0.0401 (17)	0.0188 (17)	0.0110 (13)	−0.0027 (13)	−0.0033 (13)
C4	0.0195 (13)	0.0292 (15)	0.0257 (17)	−0.0019 (11)	0.0018 (12)	0.0074 (12)
C5	0.0241 (14)	0.0258 (15)	0.0344 (19)	0.0062 (11)	0.0023 (13)	0.0022 (13)
C6	0.0255 (14)	0.0321 (15)	0.0212 (16)	0.0008 (12)	0.0034 (12)	−0.0032 (12)
C7	0.0321 (16)	0.0421 (18)	0.032 (2)	0.0049 (13)	−0.0044 (14)	0.0127 (14)
C8	0.0212 (16)	0.093 (3)	0.034 (2)	−0.0083 (17)	0.0030 (15)	−0.0152 (19)
C9	0.0248 (15)	0.0227 (14)	0.0340 (19)	0.0016 (11)	−0.0039 (13)	−0.0031 (12)
C10	0.0193 (13)	0.0219 (14)	0.0238 (16)	0.0008 (11)	−0.0006 (12)	−0.0048 (11)
C11	0.0250 (14)	0.0254 (14)	0.0252 (17)	0.0027 (11)	−0.0015 (12)	0.0026 (12)
C12	0.0225 (14)	0.0229 (14)	0.0255 (17)	−0.0025 (11)	0.0051 (12)	−0.0025 (11)
C13	0.0172 (13)	0.0271 (14)	0.0197 (16)	−0.0013 (10)	0.0016 (11)	−0.0069 (11)
C14	0.0219 (14)	0.0267 (14)	0.0202 (16)	0.0007 (11)	0.0025 (12)	0.0023 (11)
C15	0.0212 (14)	0.0194 (13)	0.0259 (17)	−0.0024 (10)	0.0033 (12)	−0.0015 (11)

### Geometric parameters (Å, °)

**Table:**

S1—O1	1.421 (2)	C7—H7A	0.9800
S1—O2	1.437 (2)	C7—H7B	0.9800
S1—N1	1.637 (2)	C7—H7C	0.9800
S1—C1	1.762 (3)	C8—H8A	0.9800
O3—N2	1.231 (3)	C8—H8B	0.9800
O4—N2	1.225 (3)	C8—H8C	0.9800
N1—C8	1.464 (4)	C9—C10	1.502 (4)
N1—C9	1.475 (3)	C9—H9A	0.9900
N2—C13	1.470 (3)	C9—H9B	0.9900
C1—C2	1.378 (4)	C10—C15	1.387 (4)
C1—C6	1.387 (4)	C10—C11	1.392 (4)
C2—C3	1.376 (4)	C11—C12	1.382 (4)
C2—H2	0.9500	C11—H11	0.9500
C3—C4	1.390 (4)	C12—C13	1.379 (4)
C3—H3	0.9500	C12—H12	0.9500
C4—C5	1.389 (4)	C13—C14	1.385 (4)
C4—C7	1.502 (4)	C14—C15	1.382 (4)
C5—C6	1.381 (4)	C14—H14	0.9500
C5—H5	0.9500	C15—H15	0.9500
C6—H6	0.9500		
			
O1—S1—O2	119.53 (15)	C4—C7—H7C	109.5
O1—S1—N1	106.68 (13)	H7A—C7—H7C	109.5
O2—S1—N1	106.66 (13)	H7B—C7—H7C	109.5
O1—S1—C1	106.68 (14)	N1—C8—H8A	109.5
O2—S1—C1	108.47 (13)	N1—C8—H8B	109.5
N1—S1—C1	108.43 (12)	H8A—C8—H8B	109.5
C8—N1—C9	113.7 (2)	N1—C8—H8C	109.5
C8—N1—S1	114.5 (2)	H8A—C8—H8C	109.5
C9—N1—S1	116.25 (17)	H8B—C8—H8C	109.5
O4—N2—O3	123.8 (2)	N1—C9—C10	112.0 (2)
O4—N2—C13	118.1 (2)	N1—C9—H9A	109.2
O3—N2—C13	118.0 (2)	C10—C9—H9A	109.2
C2—C1—C6	120.6 (3)	N1—C9—H9B	109.2
C2—C1—S1	119.8 (2)	C10—C9—H9B	109.2
C6—C1—S1	119.6 (2)	H9A—C9—H9B	107.9
C3—C2—C1	119.6 (3)	C15—C10—C11	119.3 (2)
C3—C2—H2	120.2	C15—C10—C9	120.9 (2)
C1—C2—H2	120.2	C11—C10—C9	119.8 (2)
C2—C3—C4	121.3 (3)	C12—C11—C10	121.1 (3)
C2—C3—H3	119.3	C12—C11—H11	119.5
C4—C3—H3	119.3	C10—C11—H11	119.5
C5—C4—C3	117.8 (3)	C13—C12—C11	117.9 (2)
C5—C4—C7	121.0 (3)	C13—C12—H12	121.1
C3—C4—C7	121.2 (3)	C11—C12—H12	121.1
C6—C5—C4	121.7 (3)	C12—C13—C14	122.9 (2)
C6—C5—H5	119.1	C12—C13—N2	118.3 (2)
C4—C5—H5	119.1	C14—C13—N2	118.9 (2)
C5—C6—C1	118.8 (3)	C15—C14—C13	118.1 (3)
C5—C6—H6	120.6	C15—C14—H14	121.0
C1—C6—H6	120.6	C13—C14—H14	121.0
C4—C7—H7A	109.5	C14—C15—C10	120.8 (2)
C4—C7—H7B	109.5	C14—C15—H15	119.6
H7A—C7—H7B	109.5	C10—C15—H15	119.6
			
O1—S1—N1—C8	−56.7 (3)	C2—C1—C6—C5	−0.7 (4)
O2—S1—N1—C8	174.5 (2)	S1—C1—C6—C5	177.0 (2)
C1—S1—N1—C8	57.9 (2)	C8—N1—C9—C10	66.9 (3)
O1—S1—N1—C9	167.4 (2)	S1—N1—C9—C10	−156.8 (2)
O2—S1—N1—C9	38.6 (2)	N1—C9—C10—C15	60.7 (3)
C1—S1—N1—C9	−78.0 (2)	N1—C9—C10—C11	−121.6 (3)
O1—S1—C1—C2	6.3 (3)	C15—C10—C11—C12	0.5 (4)
O2—S1—C1—C2	136.2 (2)	C9—C10—C11—C12	−177.3 (3)
N1—S1—C1—C2	−108.3 (2)	C10—C11—C12—C13	0.3 (4)
O1—S1—C1—C6	−171.4 (2)	C11—C12—C13—C14	−0.6 (4)
O2—S1—C1—C6	−41.4 (2)	C11—C12—C13—N2	179.0 (2)
N1—S1—C1—C6	74.0 (2)	O4—N2—C13—C12	7.1 (4)
C6—C1—C2—C3	1.1 (4)	O3—N2—C13—C12	−172.3 (2)
S1—C1—C2—C3	−176.6 (2)	O4—N2—C13—C14	−173.3 (2)
C1—C2—C3—C4	0.1 (5)	O3—N2—C13—C14	7.3 (4)
C2—C3—C4—C5	−1.6 (4)	C12—C13—C14—C15	0.0 (4)
C2—C3—C4—C7	176.3 (3)	N2—C13—C14—C15	−179.5 (2)
C3—C4—C5—C6	2.0 (4)	C13—C14—C15—C10	0.8 (4)
C7—C4—C5—C6	−175.9 (3)	C11—C10—C15—C14	−1.0 (4)
C4—C5—C6—C1	−0.9 (4)	C9—C10—C15—C14	176.7 (2)

### Hydrogen-bond geometry (Å, °)

**Table:**

*D*—H···*A*	*D*—H	H···*A*	*D*···*A*	*D*—H···*A*
C9—H9A···O1^i^	0.99	2.32	3.287 (4)	166

Symmetry codes: (i) *x*, *y*−1, *z*.
